# Blindness and visual impairment in Central Europe

**DOI:** 10.1371/journal.pone.0261897

**Published:** 2022-01-13

**Authors:** Marlene Glatz, Regina Riedl, Wilfried Glatz, Mona Schneider, Andreas Wedrich, Matthias Bolz, Rupert W. Strauss

**Affiliations:** 1 Department of Ophthalmology, Medical University of Graz, Graz, Austria; 2 Institute for Medical Informatics, Statistics and Documentation, Medical University of Graz, Graz, Austria; 3 Department of Ophthalmology, Kepler University Clinic of Linz, Linz, Austria; 4 Institute of Molecular and Clinical Ophthalmology, Basel, Switzerland; 5 Moorfields Eye Hospital and UCL Institute of Ophthalmology, London, United Kingdom; Save Sight Institute, AUSTRALIA

## Abstract

**Purpose:**

To assess the prevalence and causes of visual impairment and blindness in a Central European country. The findings may have implications for the planning of further research and development of therapies in order to prevent blindness.

**Setting:**

Department of Ophthalmology, Medical University of Graz, Austria.

**Design:**

Retrospective, epidemiological study.

**Methods:**

The database of the Main Confederation of Austrian Social Insurances was searched for patients with visual impairment, legal blindness or deaf-blindness. This database gathers data from patients of all insurance providers in the country who receive care due to visual impairment and blindness. To determine the prevalence of these conditions, the number of all entries recorded in February 2019 was evaluated. Additionally, all new entries between (January 1^st^,) 2017, and (December 31^st^,) 2018, were analysed for distinct characteristics, such as sex, the cause of blindness/visual impairment, and age. Since health care allowances can provide a considerable source of income (459.90€-936.90€ per month), good coverage of practically all patients who are blind and visually impaired in the country can be assumed.

**Results:**

On February 2^nd^, 2019, 17,730 patients with visual impairments, blindness or deaf-blindness were registered in Austria, resulting in a prevalence of these diagnoses of 0.2% in the country. During the observational period from 2017 to 2018, 4040 persons met the inclusion criteria. Of these, 2877 were female (65.3%), and 1527 were male (34.7%). The mean age was 75.7 ± 18.0 years (median 82). Most patients (n = 3675, 83.4%) were of retirement age, while 729 (16.6%) were working-age adults or minors. In total, an incidence of 25.0 (95% confidence limit (CL) 24.3–25.8) per 100,000 person-years was observed from 2017 to 2018. A higher incidence was observed for females (32.2, 95% CL 31.0–33.3) than for males (17.7, 95% CL 16.8–18.5). Incidences where higher for males in lower age groups (e.g. 10–14 years: rate ratio RR = 2.7, 95% CL 1.1–6.8), and higher for females in higher age groups (e.g. 70–74 years: RR = 0.6, 95% CL 0.5–0.8). In total, the most frequent diagnoses were macular degeneration (1075 persons, 24.4%), other retinal disorders (493 persons, 11.2%) and inherited retinal and choroidal diseases (IRDs) (186 persons, 4.2%). Persons with IRDs were significantly younger compared to persons with macular degeneration or retinal disorders (IRDs: median 57, range 2–96 vs 83, 5–98 and 82, 1–98 years, p<0.001). For persons of retirement age, macular degeneration, other retinal disorders and glaucoma were the three most frequent diagnoses. In contrast, among working-aged adults and children, IRDs were the leading cause of visual impairment and blindness (103 persons, 14.1%).

**Conclusion:**

These data show that IRDs are the leading cause of blindness and visual impairment in working-aged persons and children in Austria. Thus, these findings suggest to draw attention to enhance further research in the fields of emerging therapies for IRDs.

## Introduction

Children and young adults who are blind have fewer educational and employment opportunities, a lower earning potential and a poorer quality of life than those without blindness [1, 2]. An Australian study showed that individuals who are blind have a mortality rate (12/1000 person-years) seven times higher than that of the general population (1.8/1000 person-years) and that this difference is statistically significant [3]. Thus, the prevention of blindness in children is a priority within the VISION 2020 Programme of the World Health Organization (WHO) [1].

Frequency of blindness has occasionally been addressed in previous literature. A systematic review summarized various investigations about this topic in 2013 and reported the prevalence of blindness to be 0.1% in North America and 0.7% in North Africa and the Middle East [4]. In Europe, most studies have reported age-related macular degeneration (AMD) to be the main cause of blindness across all age groups [5–7], while in working-aged adults, diabetic retinopathy (DR) has been declared the leading cause in recent decades [5, 8, 9]. Interestingly, however, recent studies from England and Germany [10, 11] have reported a different perspective for this cohort over the last decade: both investigations identified inherited retinal diseases (IRDs) to be the leading cause of registered blindness for persons of working age. Epidemiologic information about blinding diseases in the European Union (EU) remains limited [12] but would be of great use, especially since the first gene therapy has been approved for use in the EU [13, 14].

One of the aims of our study is to increase awareness of the physical, psychological and financial burden caused by childhood and working-age blindness for the patients, their families and the social insurance systems. The identification of IRDs being the most frequent blinding disease in working age could possibly enhance the process of investments for research of emerging therapies and governmental planning for social care.

Austria is a central European country with approximately 8.8 million inhabitants. It has a good, inclusive national health care system, with 99% of the entire population receiving health insurance coverage (https://www.austria.org/health-care). Among the people registered at one of the welfare institutions of the Ministry for Social Affairs of Austria (MSAA), persons who are considered blind and severely visually impaired are entitled by Austrian law to a substantial health care allowance. Given the lack of data from Austria and Europe on blindness, the purpose of this study was to determine the prevalence and causes of blindness in Austrian populations.

## Methods

Based on the retrospective design and only anonymized data were obtained, the ethic commitee / institutional review board of the Medical University Graz granted an exemeption. This study was conducted in collaboration with the MSAA. A search was performed in the National Database of Care Allowance (“Pflegegeldinformationssystem”), which is managed by the Main Confederation of Austrian Social Insurances (MCASI). Every person seeking a health care allowance in Austria is centrally registered into this system, which classifies patients by case severity and includes seven grades; furthermore, all patients with disabling diseases are qualified to receive financial support depending on the severity of one or more conditions. Because the health care allowance is substantial and independent of other sources of income, good coverage of all patients who are blind and visually impaired can be assumed. An exception is persons with multiple disabilities when the other diagnoses are much more severe than the blindness or visual impairment; these patients may not appear in the database to which we had access. Based on Austrian law (§ 4a Abs. 4,5,6 Bundespflegegeldgesetz BPGG, https://www.ris.bka.gv.at/NormDokument.wxe?Abfrage=Bundesnormen&Gesetzesnummer=10008859&Artikel=&Paragraf=4a&Anlage=&Uebergangsrecht), every person with low vision is assigned to receive at least level 3 care (currently providing a health care allowance of 459.90€ per month), every person with blindness is assigned to receive level 4 care (689.80€), and every person who is both deaf and blind is assigned to receive level 5 care (936.90€).

### Definition of severe visual impairment and blindness

The level of visual impairment as assessed by visual acuity is defined according to Austrian law (BPGG) § 4a:

Visual impairment (level 3 care) is defined as visual acuity ≤ 0.05 (3/60) without visual field defects, as visual acuity ≤ 0.1 (6/60) with quadrantanopsia, as visual acuity ≤ 0.3 (6/20) with hemianopsia, or as visual acuity ≤ 1.0 (6/6) with tunnel vision.

Blindness (level 4 care) is defined as visual acuity ≤ 0.02 (1/60) without visual field defects, as visual acuity ≤ 0.03 (2/60) with quadrantanopsia, as visual acuity ≤ 0.06 (4/60) with hemianopsia, or as visual acuity ≤ 0.1 (6/60) with tunnel vision.

Deafness (in combination with blindness, level 5 care) is defined as a hearing capacity that is reduced to a degree at which verbal and other acoustic communication with the environment is impossible.

Our study cohort comprised only patients who were blind or visually impaired because of diseases that were classified as incurable by an ophthalmologist. Patients with conditions such as refractive errors are not recorded in our database.

The WHO definition of blindness is slightly less strict than the definition outlined by Austrian law and defines a visual acuity of 3/60 or worse as indicating blindness; thus, with this definition, all the patients in our study would be classified as blind.

### Cohort definition

The MSAA hosts a central database covering applications and disbursements and that collects data from different insurance carriers (the main insurance company is the MCASI. We reviewed all applications (new applications for health care allowances and requests for an increase in the level of care) from Austrian patients with a statutory diagnosis-related minimum classification of level of care for visual impairment (level of care of 3), blindness (level of care of 4) or deaf-blindness (level of care of 5) from January 1^st^, 2017, to December 31^st^, 2018. A patient is assigned to a diagnosis-related minimum classification for the respective ophthalmic condition when he or she has no additional impairments that would require higher levels of care; assessments are carried out by certified general physicians including the acknowledgment of medical certifications by corresponding specialists. Since coding according to the 10^th^ version of the international classification of diseases (ICD-10) was not fully implemented in the central database until 2017, we restricted our observational period to the years 2017 and 2018. Implausible data entries regarding diagnosis-related minimum classifications of level of care were excluded. All data analysed in this study were provided by the MSAA after the removal of any personal identifiable information (names, date of birth, etc.).

The following parameters were evaluated: sex, level of care, federal state of residence, type of application (first application or application for a higher level of care), date of application, level of impairment, ICD-10 diagnosis, age at the date of application, and history of occupational accidents. The following groups were defined according to the age at the time of application: persons in retirement (men aged ≥ 65 years and women aged ≥60), working-aged adults (18–59/64 years) and minors/children (<18 years). The diagnoses were grouped as follows: congenital and developmental diseases (malformations, retinopathy of prematurity (ROP), amblyopia), corneal disorders, DR, diseases of the lens, disorders of the optic nerve and visual pathways, glaucoma, IRDs, inflammation of the eye, macular degeneration, myopia, neoplasms, other retinal disorders, retinal detachment and diseases of the vitreous body, and others.

The MCASI dataset includes exactly one main ICD-10 diagnosis per case. Since the main purpose of the database is to reveal information that is especially relevant for nursing care rather than medical care, the nursing-relevant main diagnosis (as assessed by a general physician serving as a judicially certified expert) might not necessarily be the diagnosis that leads to a designation of blindness. In such cases, when only nonophthalmic diagnoses were available, we considered the diagnosis to be “missing” in our study. Cases of visual impairment/blindness were coded on the basis of the most recent version of the WHO ICD-10 classification system (https://www.who.int/classifications/icd/icdonlineversions/en/).

Our data included both entries for new applications for health care allowances and applications for an increase in the level of care. The worsening of a medical condition can be associated with an increase in the level of care, and when the corresponding criteria (see above) are fulfilled, patients are eligible to reapply for the next level and receive more financial support. A visually impaired person (level 3 care) could potentially reapply for two higher levels of care (i.e., level 4 = blindness and level 5 = deaf-blindness); hence, a person can theoretically be listed up to three times, although this issue was extremely unlikely. In order to estimate the possibility of double entries, we explored a sample of all 729 children and working-aged adults with the following rationale: A double entry must fulfill the following criteria: it must be an application for elevation. There are some parameters that would stay the same for this patient: sex, disease, age (the same or maximally 2 years older Applying these criteria to a sample of all 729 children and working-aged adults, we could not rule out the possibility for double entry in only 12 cases (of note: those are not cases of double entries but only those for that we could not definitely rule out this possibility). Therefore, we analysed all the data, assuming that the duplicates within the short period of time we analysed did not affect the distributions of the characteristics of our cases.

On February 2^nd^, 2019, the MCASI reported the number of all patients receiving social care allowances because of diagnosis-related minimum classifications of visual impairment, blindness and deaf-blindness, which allowed us to estimate the prevalence of those conditions in Austria.

In statistical analyses, continuous parameters are presented as the mean ± standard deviation or the median and range (minimum-maximum), and categorical data are presented as frequency and percentage. Age at time of application was compared between male and females by using Mann–Whitney U tests and between the most frequent diagnoses by Kruskal-Wallis test. Incidence was calculated per 100,000 person-years. The age-, sex- and province-specific incidences were calculated based on the Austrian populations in 2017 and 2018. The rate ratios and their corresponding 95% confidence limits (CLs) for females and males were calculated within age groups (0–4 years, 5–9 years up to 95 years and older). The population data for the estimation of incidence were retrieved from the central official institute for statistics (Statistik Austria; https://www.statistik.at/web_en/statistics/PeopleSociety/population/index.html; last accessed July 9th, 2020).

The study was conducted according to the International Council for Harmonisation of Technical Requirements for Pharmaceuticals for Human Use (ICH) Good Clinical Practice (GCP) Guidelines, the applicable regulatory requirements and the current version of the Declaration of Helsinki [15].

## Results

### All age groups

On February 2^nd^, 2019, 17,730 patients had a diagnosis-related minimum classification of visual impairment, blindness or deaf-blindness in Austria. In that month, the Austrian population included 8,877,637 people. (https://www.statistik.at/web_de/statistiken/menschen_und_gesellschaft/bevoelkerung/index.html). The prevalence of diagnosis-related minimum classifications of blindness, visual impairment, or deaf-blindness in Austria was therefore 0.2% at this time.

The data included 5040 applications for a health care allowance (new applications and applications for elevation) for visual impairment, blindness or deaf-blindness. For analysis, 636 entries were excluded either due to an application date outside the observational period (2017–2018; n = 424) or implausible deviations from the diagnosis-related minimum classifications of level of care (n = 212).

The remaining 4404 patients (2877 female (65.3%) and 1527 male (34.7%)) met the inclusion criteria. In 2017, there were 2434 (55.3%) applications, and in 2018, there were 1970 (44.7%); 2697 (61.2%) patients had visual impairment, 1693 (38.4%) were blind and 14 (0.3%) were deaf-blind. No persons were blind or visually impaired because of an occupational accident. A total of 2338 (53.1%) applications were new applications, and 2066 (46.9%) were applications for a higher level of care.

**Fig 1:**
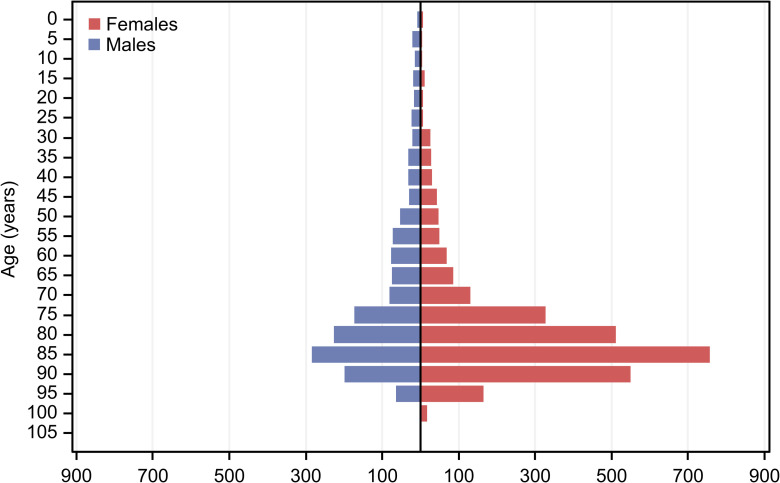
Age distribution for males and females among all patients who requested health care allowances in 2017 and 2018 in Austria because of a diagnosis-related minimum classification of visual impairment, blindness or deaf-blindness.

The mean age was 75.7 ± 18.0 years (median 82, range 0–103 years, Fig 1). Most patients (n = 3675, 83.4%) were of retirement age, and 729 patients (16.6%) were working-age adults or minors. The women were significantly older than the men at the time of the application (women: median 83, range 0–103; men: median 78, range 0–99; p<0.001, Mann–Whitney U test).

Age- and sex-specific incidences were calculated based on the Austrian populations in 2017 and 2018. In total, an incidence of 25.03 (95% CL 24.3–25.8) per 100,000 person-years was observed over the years 2017 and 2018. A higher incidence was observed for females (32.2, 95% CL 31.0–33.3) than for males (17.7, 95% CL 16.8–18.5). Within age subgroups, the incidence was higher for young males than for young females (age groups 5–9 and 10–14 years) and higher for females than for males in the 70–89-year age group (Table 1). The incidences for the individual Austrian federal states are presented in Table 2, varying between 17.5 (95% CL 16.0–19.0) per 100,000 person-years in Upper Austria and 37.9 (95% CL 34.3–41.5) per 100,000 person-years in Carinthia.

**Table 1:** Age- and sex-specific incidences per 100,000 person-years among all patients who requested a health care allowance in 2017 and 2018 in Austria because of a diagnosis-related minimum classification of visual impairment, blindness and deaf-blindness (source of reference population: STATISTIK AUSTRIA, https://www.statistik.at/web_de/statistiken/menschen_und_gesellschaft/bevoelkerung/index.html, accessed May 17^th^, 2018) (bold values: Statistically significant results).

Age	Number of cases		Incidence per 100,000 person years		Rate Ratio	95% Confidence Limits	
	Male	Female	Male	Female			
0–4 years	19	8	4.3	1.9	2.23	0.98	5.10
5–9 years	19	7	4.4	1.7	**2.56**	**1.08**	**6.09**
10–14 years	17	6	3.9	1.5	**2.69**	**1.06**	**6.83**
15–19 years	16	8	3.4	1.9	1.84	0.79	4.29
20–24 years	19	9	3.4	1.7	1.99	0.90	4.39
25–29 years	22	12	3.6	2.0	1.75	0.87	3.54
30–34 years	25	25	4.1	4.2	0.97	0.56	1.69
35–39 years	33	32	5.6	5.6	1.01	0.62	1.65
40–44 years	23	29	4.0	5.0	0.80	0.46	1.38
45–49 years	42	44	6.2	6.5	0.96	0.63	1.46
50–54 years	62	54	8.6	7.6	1.14	0.79	1.64
55–59 years	70	56	11.1	8.8	1.26	0.89	1.79
60–64 years	72	71	14.5	13.4	1.08	0.78	1.50
65–69 years	92	99	21.6	20.8	1.04	0.78	1.38
70–74 years	97	184	28.3	45.1	**0.63**	**0.49**	**0.80**
75–79 years	216	429	65.2	102.1	**0.64**	**0.54**	**0.75**
80–84 years	246	581	145.6	230.5	**0.63**	**0.54**	**0.73**
85–89 years	269	743	266.1	397.2	**0.67**	**0.58**	**0.77**
90–94 years	145	395	426.9	411.6	1.04	0.86	1.25
95 years and older	23	85	400.5	354.7	1.13	0.71	1.79

**Table 2:** Geographical locations of all patients who requested a health care allowance in 2017 and 2018 in Austria because of a diagnosis-related minimum classification of visual impairment, blindness and deaf-blindness.

District	Patients during the 2-year study period	Population 2017	Population 2018	Rate per 100,000 person years		
				Rate	95% Confidence Limits	
Vienna	964 (21.9%)	1 883 706	1 893 779	25.5	23.9	27.1
Lower Austria	878 (19.9%)	1 669 944	1 677 104	26.2	24.5	28.0
Burgenland	167 (3.8%)	292 592	293 490	28.5	24.2	32.8
Upper Austria	518 (11.8%)	1 472 422	1 481 298	17.5	16.0	19.0
Styria	589 (13.4%)	1 239 153	1 242 635	23.7	21.8	25.6
Carinthia	425 (9.7%)	560 915	561 030	37.9	34.3	41.5
Salzburg	300 (6.8%)	551 863	554 766	27.1	24.0	30.2
Tyrol	354 (8.0%)	749 853	753 397	23.5	21.1	26.0
Vorarlberg	169 (3.8%)	391 334	393 918	21.5	18.3	24.8
Unknown	40 (0.9%)					

The diagnoses of the patients varied with age. Overall, the most frequent diagnoses were macular degeneration, other retinal disorders and IRDs (Table 3). Age at time of application differs significantly for those three diagnoses with mean ages of 81.1 ± 11.3 years (median 83, range 5–98) for macular degeneration, 77.6 ± 16.1 years (median 82, range 1–98) for other retinal disorders and 56.2 ± 25.1 years (median 57, range 2–96) for IRDs (p<0.001, Kruskal-Wallis test). For persons of the age of retirement, macular degeneration, other retinal disorders and glaucoma were the three most frequent causes of the diagnoses. In contrast, for working-aged adults and children, IRDs were the leading causes (Table 3).

**Table 3:** Number and percentage of all patients who requested a health care allowance in 2017 and 2018 in Austria because of a diagnosis-related minimum classification of visual impairment, blindness or deaf-blindness according to the diagnosis, age and sex (M = male, F = female, most frequent diseases bold and underlined).

	All			0-retirement			0–17			18-retirement			Retirement		
diagnosis	Total	M	F	Total	M	F	Total	M	F	Total	M	F	Total	M	F
congenital and developmental diseases (malformation ROP, amblyopia)	102 2.3%	43 2.8%	59 2.1%	38 5.2%	28 6.4%	10 3.4%	20 21.7%	14 21.9%	6 21.4%	18 2.8%	14 3.7%	4 1.5%	64 1.7%	15 1.4%	49 1.9%
corneal disorders	39 0.9%	15 1.0%	24 0.8%	17 2.3%	9 2.1%	8 2.8%	1 1.1%	1 3.6%	1 3.6%	16 2.5%	9 2.4%	7 2.7%	22 0.6%	6 0.6%	16 0.6%
diabetic retinopathy	101 2.3%	49 3.2%	52 1.8%	29 4.0%	20 4.6%	9 3.1%				29 4.6%	20 5.3%	9 3.4%	72 2.0%	29 2.7%	43 1.7%
diseases of the lens	27 0.6%	11 0.7%	16 0.6%	5 0.7%	4 0.9%	1 0.3%				5 0.8%	4 1.1%	1 0.4%	22 0.6%	7 0.6%	15 0.6%
**disorders of the optic nerve and visual pathways**	126 2.9%	71 4.6%	55 1.9%	70 9.6%	45 10.3%	25 8.6%	8 8.7%	7 10.9%	1 3.6%	62 9.7%	38 10.1%	24 9.2%	56 1.5%	26 2.4%	30 1.2%
**glaucoma**	139 3.2%	59 3.9%	80 2.8%	20 2.7%	15 3.4%	5 1.7%				20 3.1%	15 4.0%	5 1.9%	119 3.2%	44 4.0%	75 2.9%
**IRDs**	186 4.2%	79 5.2%	107 3.7%	103 14.1%	62 14.1%	41 14.1%	18 19.6%	10 15.6%	8 28.6%	85 13.3%	52 13.9%	33 12.6%	83 2.3%	17 1.6%	66 2.6%
inflammation of the eye	70 1.6%	15 1.0%	55 1.9%	7 1.0%	2 0.5%	5 1.7%				7 1.1%	2 0.5%	5 1.9%	63 1.7%	13 1.2%	50 1.9%
**macular degeneration**	1075 24.4%	294 19.3%	781 27.1%	49 6.7%	24 5.5%	25 8.6%	7 7.6%	4 6.3%	3 10.7%	42 6.6%	20 5.3%	22 8.4%	1026 27.9%	270 24.8%	756 29.2%
myopia	11 0.2%	6 0.4%	5 0.2%	5 0.7%	4 0.9%	1 0.3%				5 0.8%	4 1.1%	1 0.4%	6 0.2%	2 0.2%	4 0.2%
neoplasms	46 1.0%	21 1.4%	25 0.9%	15 2.1%	11 2.5%	4 1.4%	4 4.3%	3 4.7%	1 3.6%	11 1.7%	8 2.1%	3 1.1%	31 0.8%	10 0.9%	21 0.8%
**other retinal disorders**	493 11.2%	152 10.0%	341 11.9%	59 8.1%	28 6.4%	31 10.7%	8 8.7%	7 10.9%	1 3.6%	51 8.0%	21 5.6%	30 11.5%	434 11.8%	124 11.4%	310 12.0%
others	84 1.9%	36 2.4%	48 1.7%	17 2.3%	8 1.8%	9 3.1%	2 2.2%	1 1.6%	1 3.6%	15 2.4%	7 1.9%	8 3.1%	67 1.8%	28 2.6%	39 1.5%
retinal detachment and diseases of the vitreous body	21 0.5%	7 0.5%	14 0.5%	8 1.1%	3 0.7%	5 1.7%	2 2.2%	2 3.1%	2 3.1%	6 0.9%	1 0.3%	5 1.9%	13 0.4%	4 0.4%	9 0.3%
missing or unknown	1884 42%	669 43%	1215 42%	287 39%	176 40%	111 38%	22 23%	16 25%	6 21%	265 41%	160 42%	105 40%	1597 43%	493 45%	1104 42%

### Special interest cohort: Children and working-age adults

The cohort of children and working-age adults (n = 729) consisted of 290 (39.8%) females and 439 (60.2%) males. The mean age of all newly registered persons was 41.6 ± 16.8 years (median 47, range 0–64) in this cohort. Within patients <60 years of age, females were significantly older than males (41.5 ± 14.4 years, median 46, range 1–59 versus 37.8 ± 17.4 years, median 42, range 0–59 for patients < 60 years, p = 0.033, Mann–Whitney U test). From the 729 persons, 410 (56.2%, male: n = 251, female: n = 159) were visually impaired, 316 (43.3%, male: n = 186, female: n = 130) were blind and 3 (0.4%, male: n = 2, female: n = 1) were deaf-blind. A total of 537 (73.7%) applications were new applications, and 192 (26.3%) were applications for a higher level of care.

As mentioned above, IRDs were the leading causes of visual impairment and blindness in this cohort, followed by disorders of optic nerve/visual pathways and other retinal disorders. A separate sex analysis showed the same distribution as described above for males, whereas females most often suffered from IRDs, followed by other retinal disorders and disorders of the optic nerve and visual pathways and macular degeneration. For both men and women together, DR was the sixth most frequent diagnosis in this cohort (Table 3, 0-retirement group).

For working-aged adults (18 years-retirement) (n = 637), the most frequent diagnosis was IRDs. For children, the most frequent diagnoses were congenital and developmental diseases, followed by IRDs (Table 3). The age distributions for IRDs for men (mean age 37.1 ± 15.9) and women (mean age 37.5 ± 16.9) were similar. Deaf-blindness occurred in only 3 children and working-aged adults, who were over 40 years of age. Finally, for our core cohort, missing diagnoses were more frequent in men (176, 61.3%) than in women (111, 38.6%), and these numbers increased with age.

## Discussion

As reported by the WHO Universal Eye Health Action Plan and the European Society of Ophthalmology’s Pilot Committee on Public Health [16], only a few studies on the prevalence, incidence and causes of blindness in Europe have been conducted thus far, and therefore, there remains a paucity of information on the causes of blindness from central Europe [12]. A distinctive observation of working-aged persons has only marginally been conducted in the recent literature [11, 17].

While blindness is more common among retirees, quality of life is affected even more in younger persons [18–21]: the affected lifespan is much longer, the corresponding ophthalmological problems are more often of greater importance or concern to the patient, and the patient can likely regain complete independence and mobility if the blindness could be resolved; as a consequence, these individuals participate less in the employment market [22] and even in higher education. A Danish investigation suggested that at the age of 40 years, fewer patients with generalized retinal dystrophy than controls had a high income, a high educational level and were married, and more patients than controls were already pensioners at this relatively young age [2]. The aim of our study was to investigate the main causes of visual impairment/blindness, especially in working-aged persons and children in Austria, in order to provide information on how to improve potential preventative and therapeutic measures. The reports of Liew et al. and Finger et al. [10, 11] led us to consider whether DR, although commonly known to be the major cause of visual impairment or blindness in the Western world so far [5, 8, 9], is still the leading cause of blindness in working-aged people in Austria. To the best of our knowledge, there have been no reports on this topic, and until 2012, potential information was not centrally acquired in this country as it is now at the MSAA.

In accordance with previous reports, our results show that AMD is the overall leading cause of blindness in European countries, especially in the United Kingdom (UK), including England and Wales [5] and Scotland [6, 7], as well as in Ireland [23, 24] and Italy [25]. Other leading causes of blindness in Europe include glaucoma, IRDs and cerebrovascular disease in Scotland [6], glaucoma and retinitis pigmentosa in Ireland [24], cataracts and glaucoma in Italy [25] and untreated cataracts and glaucoma in Moldova [26]. Outside of the European region, in countries with a comparable socioeconomic status, the findings are similar: the most frequent causes of legal blindness are AMD, glaucoma and myopic maculopathy in Israel [27], AMD, glaucoma and cataracts in the United States [28] and AMD, glaucoma and DR in Australia [29].

Furthermore, various studies have reported refractive errors to be a frequent cause of blindness [12, 30]. Though every habitant has easy access to social insurances, and additionally, there are a number of routine investigations for every child at schools and for every 18 year old male by the federal army in Austria, there are still some few patients who are not provided with corresponding correcting glasses or contact lenses without these mandatory examinations. However, those are not included in our study, because they do not qualify for care allowance. For care allowance, a medical certificate by an ophthalmologist is required, which clearly states an incurable blinding condition or visual impairment. Therefore, the assessment of best-corrected visual acuity is a mandatory step prior being qualified for care allowance.

Regarding working-aged individuals, decades of scientific research have suggested that DR is the leading cause of blindness [8, 9, 31, 32]. Exhaustive scientific research and reorganization of health care processes such as the NHS Screening Programme in the UK have been conducted. In Austria, annual screening by an ophthalmologist is currently provided for every patient with diabetes with or without mild DR, while patients with severe forms of DR are examined more frequently (https://www.oedg.at/pdf/OEDG_Pocket_Guide_2019-07.pdf). Programs for prevention and new therapies, such as intravitreal injections (anti-vascular endothelial growth factor (VEGF) or corticosteroids), are included in routine care [33]. Under these circumstances, blinding DR has become rarer in working-age people, while IRDs are now the leading cause of blindness in the UK (11), Germany [10] and, according to our results, also Austria. This is in agreement with results from Ireland, where a study reported retinitis pigmentosa to be the leading cause of blindness in working-aged individuals already in 1998 [23].

Hence, while improved treatment options for DR have lowered the incidence of visual impairment and blindness and caused IRDs to be the leading cause of these issues today, our findings and those of the aforementioned studies have several implications. First, awareness must been raised among (general) ophthalmologists to better understand and address IRDs, especially in respect to emerging therapies, and also that genetic testing due to the gene-dependent approach of not only gene augmentation but also pharmacotherapy is mandatory [34]. Second, these data are clear arguments to focus research on IRDs, also from a funding perspective. Third, from an economic point of view, e.g., for social insurance, although approved or emerging therapies such as gene augmentation therapy are very expensive, the therapeutic benefits may not only reduce the personal burden of patients but also help reduce costs for medical care and allow patients to participate in the employment market [21, 35]. Interestingly, only three groups of diseases are responsible for half of all certifications for working-aged people in Europe, as our study and those conducted in Germany and the UK have shown: IRDs, diabetic retinopathy/other retinal disorders and glaucoma/diseases of optic nerve and visual pathways [11, 12].

Another mentionable finding of our study is how the sex distribution varies with age. Since the extent of these differences was so striking and since our groups (children, working-aged adults and children) are defined by economic activity rather than the age (retirement age is the age of 60 years for females and 65 years for males in Austria) we created an additional table that carves out differences of sex distribution across age groups so that our data may be even better comparable for other researchers with other cohort definitions (Table 1). Taking all age groups together, in accordance with the results of previous studies [10, 36], the prevalence of blindness is higher in women than in men overall and for nearly all disease categories. However, the sex distribution was completely different for patients under 30 years of age: in this group, more males were affected. For the 5–14-years age groups, this difference was also statistically significant (Table 1: Frequency Distribution). The predominance of males in younger age groups has rarely been reported, but detailed observations of the results of other investigations also show some evidence of this finding [7, 9, 10].

High myopia was a larger issue in some other studies, with incidences of 2.8% [11], 5% [10] and 11.8% [37] versus the 0.2% seen in our study. This observation may be related to the use of different codes; some of the retinal detachments in our cohort could possibly be seen as consequences of high myopia but were coded as myopia in other reports. Additionally, some myopic retinopathies and maculopathies could have been coded as “other retinal disorders” in our study. However, myopic changes, such as retinal holes, are commonly detected and treated very early in Austria,; a referral from general practitioners to see an ophthalmologist is–in contrast e.g. to the UK—not needed, and “medical ophthalmology” is covered by ophthalmologists rather than optometrists, which are rarely know so far in Austria. Thus, a thorough and regular routine retinal examination is typically performed in myopic patients.

Our data revealed only a small variation in blindness/visual impairment cases by geographical location, though from an economical and geographical point of view, there are large differences between Eastern federal states (e.g. Vienna) and Western federal states that are largely dependent on rural income sources such as tourism (skiing, hiking etc.) and agriculture. In contrast, a study conducted in England [38] described an almost 11-fold higher level of variation. An explanation for this inconsistency could be the differences in the inhabitant/area ratio and the fact that Austria provides an excellent and easily accessible health care system with small and good hospitals reachable within a short travelling distance, even from rural areas.

A strength of our study is that data from centralized registers were evaluated. Austria is a rather small country, but in contrast to the challenges researchers from other countries may face, where the incentive for registration is lower [39], our data are quite representative and complete since the financial support that is allowed with registration is substantial.

A limitation of our study is that persons with multiple impairments and very high levels of care are not identifiable in our database after they are diagnosed with blindness. We therefore suspect that, especially over the age of retirement, there are persons with multiple impairments that might not appear in our data base, so that the rate of blindness/visual impairment might be even higher than we could verify in this survey. However, for the patients identified herein, the ophthalmological impairment/burden is the leading problem of affecting their lives by being their “leading” diagnosis to be eligible for financial care allowance. A second limitation of our study is that there were patients registered with “unknown diseases”. Only the main diagnosis was documented in the database, and ICD-10 coding was performed by general practitioners (GPs) based on ophthalmologic clinical reports. All patients with blindness/visual impairment but no other disease that is legally more severe than the blindness/visual impairment were registered in the database. However, when the GPs considered other diseases more relevant for care allowance (e.g., impaired mobility or psychological diseases), they potentially might have “overruled” the ophthalmologic diagnosis via ICD-10 coding, even if correct from the “legal”, administrative point of view, this diagnosis might be less severe in daily life. Additionally, in some cases, instead of specific codes, general codes such as “visual impairment” or even “visual disturbances” were chosen. Other authors from the few available relevant studies faced similar issues, i.e., the rate of general codes or unknown diagnoses ranged from 10.9% [11] to 15.6% [7] and 19.5% [10]. It is presumable, however, that the complete and accurate coding of cases would not have changed the key results. Regarding the completely unknown diseases, it is suspected that these diseases are not mainly DRs; since diabetes (including mild forms) is so prevalent in Austria (6–7%, https://www.diabetes.or.at/fileadmin/Dokumente/Aktuelles/2017_oest_diabetesbericht.pdf), GPs may not always assign the correct ICD-10 codes. Patients with DR, however, could be among the patients who have multiple impairments but are not listed in the database as patients with ophthalmological diagnoses. For example, these patients could have severely impaired mobility affecting their daily lives even more than blindness. On the other hand, this might be also applicable for e.g. syndromic retinitis pigmentosa cases (with the exception of Usher syndromes which are highly likely coded as “deaf-blind”). Some DRs (but also some IRDs) could be included in the group of diagnoses that was coded as “other retinal disorders” with no additional information. However, even if all data from this group (51 working-aged individuals) were added to the group of DPs (thus totalling 67), they would still be rarer than IRDs (85). Additionally, in 19 persons aged 40 years or younger, the diagnosis of “macular degeneration” was given. These cases must have been considered additional cases of IRDs.

In this dataset, we cannot rule out the possibility of double entries due to applications for a higher level of care. However, a manual check by an expert ophthalmologist was performed on all data from working-aged persons and showed that few double entries appeared.

Our data may suggest that rigorous efforts to reduce blindness caused by DR in recent decades could have been effective. Currently, in our country, IRDs are the most important problem for persons of working age. This implicates that future studies should also focus on the research and development of therapies for IRDs, in order to treat individual cases and prevent long-term visual impairment or blindness in young and working-aged patients.

## References

[1] Gilbert C, Awan H. Blindness in children. BMJ (Clinical research ed). 2003;327(7418):760–1. 10.1136/bmj.327.7418.760 PMC214052 14525849

[2] Bertelsen M, Linneberg A, Rosenberg T. Socio-economic characteristics of patients with generalized retinal dystrophy in Denmark. Acta Ophthalmol. 2015;93(2):134–40. doi: 10.1111/aos.12467 24953749

[3] Crewe JM, Morlet N, Morgan WH, Spilsbury K, Mukhtar AS, Clark A, et al. Mortality and hospital morbidity of working-age blind. Br J Ophthalmol. 2013;97(12):1579–85. doi: 10.1136/bjophthalmol-2013-303993 24123905

[4] Stevens GA, White RA, Flaxman SR, Price H, Jonas JB, Keeffe J, et al. Global prevalence of vision impairment and blindness: magnitude and temporal trends, 1990–2010. Ophthalmology. 2013;120(12):2377–84. doi: 10.1016/j.ophtha.2013.05.025 23850093

[5] Bunce C, Wormald R. Causes of blind certifications in England and Wales: April 1999-March 2000. Eye (London, England). 2008;22(7):905–11. doi: 10.1038/sj.eye.6702767 17332762

[6] Quartilho A, Simkiss P, Zekite A, Xing W, Wormald R, Bunce C. Leading causes of certifiable visual loss in England and Wales during the year ending 31 March 2013. Eye (London, England). 2016;30(4):602–7. doi: 10.1038/eye.2015.288 26821759 PMC5108547

[7] Bamashmus MA, Matlhaga B, Dutton GN. Causes of blindness and visual impairment in the West of Scotland. Eye (London, England). 2004;18(3):257–61. doi: 10.1038/sj.eye.6700606 15004574

[8] Mohamed Q, Gillies MC, Wong TY. Management of diabetic retinopathy: a systematic review. Jama. 2007;298(8):902–16. doi: 10.1001/jama.298.8.902 17712074

[9] Rosenberg T, Klie F. Current trends in newly registered blindness in Denmark. Acta ophthalmologica Scandinavica. 1996;74(4):395–8. doi: 10.1111/j.1600-0420.1996.tb00716.x 8883558

[10] Finger RP, Fimmers R, Holz FG, Scholl HP. Prevalence and causes of registered blindness in the largest federal state of Germany. Br J Ophthalmol. 2011;95(8):1061–7. doi: 10.1136/bjo.2010.194712 21378005

[11] Liew G, Michaelides M, Bunce C. A comparison of the causes of blindness certifications in England and Wales in working age adults (16–64 years), 1999–2000 with 2009–2010. BMJ Open. 2014;4(2):e004015. doi: 10.1136/bmjopen-2013-004015 24525390 PMC3927710

[12] Bourne RR, Stevens GA, White RA, Smith JL, Flaxman SR, Price H, et al. Causes of vision loss worldwide, 1990–2010: a systematic analysis. Lancet Glob Health. 2013;1(6):e339–49. doi: 10.1016/S2214-109X(13)70113-X 25104599

[13] Kellner U, Renner AB, Herbst SM, Kellner S, Weinitz S, Weber BH. [Hereditary retinal dystrophies]. Klin Monbl Augenheilkd. 2012;229(2):171–93; quiz 94–6. doi: 10.1055/s-0031-1280461 22241577

[14] Gruntman AM, Flotte TR. The rapidly evolving state of gene therapy. FASEB journal: official publication of the Federation of American Societies for Experimental Biology. 2018;32(4):1733–40. doi: 10.1096/fj.201700982R 31282760

[15] World Medical Association Declaration of Helsinki: ethical principles for medical research involving human subjects. Jama. 2013;310(20):2191–4. doi: 10.1001/jama.2013.281053 24141714

[16] Németh J, Tóth G, Resnikoff S, de Faber JT. Preventing blindness and visual impairment in Europe: What do we have to do? European journal of ophthalmology. 2019;29(2):129–32. doi: 10.1177/1120672118819397 30572715

[17] Flaxman SR, Bourne RRA, Resnikoff S, Ackland P, Braithwaite T, Cicinelli MV, et al. Global causes of blindness and distance vision impairment 1990–2020: a systematic review and meta-analysis. Lancet Glob Health. 2017;5(12):e1221–e34. doi: 10.1016/S2214-109X(17)30393-5 29032195

[18] Crewe JM, Morlet N, Morgan WH, Spilsbury K, Mukhtar A, Clark A, et al. Quality of life of the most severely vision-impaired. Clin Exp Ophthalmol. 2011;39(4):336–43. doi: 10.1111/j.1442-9071.2010.02466.x 21070550

[19] Wong EY, Chou SL, Lamoureux EL, Keeffe JE. Personal costs of visual impairment by different eye diseases and severity of visual loss. Ophthalmic Epidemiol. 2008;15(5):339–44. doi: 10.1080/09286580802227394 18850471

[20] Berdeaux G, Brézin AP, Fagnani F, Lafuma A, Mesbah M. Self-reported visual impairment and mortality: a French nationwide perspective. Ophthalmic Epidemiol. 2007;14(2):80–7. doi: 10.1080/09286580600899691 17464855

[21] Chaumet-Riffaud AE, Chaumet-Riffaud P, Cariou A, Devisme C, Audo I, Sahel JA, et al. Impact of Retinitis Pigmentosa on Quality of Life, Mental Health, and Employment Among Young Adults. Am J Ophthalmol. 2017;177:169–74. doi: 10.1016/j.ajo.2017.02.016 28237413

[22] Chakravarthy U, Biundo E, Saka RO, Fasser C, Bourne R, Little JA. The Economic Impact of Blindness in Europe. Ophthalmic Epidemiol. 2017;24(4):239–47. doi: 10.1080/09286586.2017.1281426 28665742

[23] Munier A, Gunning T, Kenny D, O’Keefe M. Causes of blindness in the adult population of the Republic of Ireland. Br J Ophthalmol. 1998;82(6):630–3. doi: 10.1136/bjo.82.6.630 9797662 PMC1722638

[24] Kelliher C, Kenny D, O’Brien C. Trends in blind registration in the adult population of the Republic of Ireland 1996–2003. Br J Ophthalmol. 2006;90(3):367–71. doi: 10.1136/bjo.2005.075861 16488964 PMC1856962

[25] Cruciani F, Abdolrahimzadeh S, Vicari A, Amore FM, Di Pillo S, Mazzeo L. Causes of blind certification in an Italian province and comparison with other European countries. La Clinica terapeutica. 2010;161(1):e11–6. 20544148

[26] Zatic T, Bendelic E, Paduca A, Rabiu M, Corduneanu A, Garaba A, et al. Rapid assessment of avoidable blindness and diabetic retinopathy in Republic of Moldova. Br J Ophthalmol. 2015;99(6):832–6. doi: 10.1136/bjophthalmol-2014-305824 25550353

[27] Avisar R, Friling R, Snir M, Avisar I, Weinberger D. Estimation of prevalence and incidence rates and causes of blindness in Israel, 1998–2003. The Israel Medical Association journal: IMAJ. 2006;8(12):880–1. 17214111

[28] Congdon N, O’Colmain B, Klaver CC, Klein R, Muñoz B, Friedman DS, et al. Causes and prevalence of visual impairment among adults in the United States. Arch Ophthalmol. 2004;122(4):477–85. doi: 10.1001/archopht.122.4.477 15078664

[29] Yong VK, Morgan WH, Cooper RL, Shaw M, Bremner AP, Bulsara M, et al. Trends in registered blindness and its causes over 19 years in Western Australia. Ophthalmic Epidemiol. 2006;13(1):35–42. doi: 10.1080/09286580500473779 16510345

[30] Bourne RRA, Jonas JB, Bron AM, Cicinelli MV, Das A, Flaxman SR, et al. Prevalence and causes of vision loss in high-income countries and in Eastern and Central Europe in 2015: magnitude, temporal trends and projections. Br J Ophthalmol. 2018;102(5):575–85. doi: 10.1136/bjophthalmol-2017-311258 29545417 PMC5909755

[31] Cheung N, Mitchell P, Wong TY. Diabetic retinopathy. Lancet. 2010;376(9735):124–36. doi: 10.1016/S0140-6736(09)62124-3 20580421

[32] Zheng Y, He M, Congdon N. The worldwide epidemic of diabetic retinopathy. Indian journal of ophthalmology. 2012;60(5):428–31. doi: 10.4103/0301-4738.100542 22944754 PMC3491270

[33] Wong TY, Sun J, Kawasaki R, Ruamviboonsuk P, Gupta N, Lansingh VC, et al. Guidelines on Diabetic Eye Care: The International Council of Ophthalmology Recommendations for Screening, Follow-up, Referral, and Treatment Based on Resource Settings. Ophthalmology. 2018;125(10):1608–22. doi: 10.1016/j.ophtha.2018.04.007 29776671

[34] Scholl HP, Strauss RW, Singh MS, Dalkara D, Roska B, Picaud S, et al. Emerging therapies for inherited retinal degeneration. Science translational medicine. 2016;8(368):368rv6. doi: 10.1126/scitranslmed.aaf2838 27928030

[35] Galvin O, Chi G, Brady L, Hippert C, Del Valle Rubido M, Daly A, et al. The Impact of Inherited Retinal Diseases in the Republic of Ireland (ROI) and the United Kingdom (UK) from a Cost-of-Illness Perspective. Clinical ophthalmology (Auckland, NZ). 2020;14:707–19. 10.2147/OPTH.S241928 PMC7062501 32184557

[36] Abou-Gareeb I, Lewallen S, Bassett K, Courtright P. Gender and blindness: a meta-analysis of population-based prevalence surveys. Ophthalmic Epidemiol. 2001;8(1):39–56. doi: 10.1076/opep.8.1.39.1540 11262681

[37] Cruciani F, Amore F, Albanese G, Anzidei R. Investigation about causes of blindness and low vision among members of Blind and Visually Impaired Italian Union (UICI). La Clinica terapeutica. 2011;162(2):e35–42. 21533307

[38] Malik AN, Bunce C, Wormald R, Suleman M, Stratton I, Gray JA. Geographical variation in certification rates of blindness and sight impairment in England, 2008–2009. BMJ Open. 2012;2(6). 10.1136/bmjopen-2012-001496 PMC3532990 23166126

[39] Barry RJ, Murray PI. Unregistered visual impairment: is registration a failing system? Br J Ophthalmol. 2005;89(8):995–8. doi: 10.1136/bjo.2004.059915 16024852 PMC1772775

