# Correction: Blindness and visual impairment in Central Europe

**DOI:** 10.1371/journal.pone.0335487

**Published:** 2025-10-28

**Authors:** The PLOS One Editors

## Notice of Republication

This article [1] was republished on September 17, 2025, to address an issue identified post-publication. Please download this article again to view the correct version.

The article’s Data Availability statement is updated to: Due to legal restrictions in accordance with the national data protection ordinance, the Main Confederation of Austrian Social Insurances/ Ministry of Social Affairs of Austria, the raw data underlying this publication are available only upon request. Interested and qualified researchers who meet the criteria for access to confidential data may submit data access requests to: Main Confederation of Austrian Social Insurances and the Ministry of Social Affairs of Austria Sozialministerium/ Service für Bürgerinnen und Bürger Stubenring 1, 1010 Wien (PosteingangAllgemein@sozialversicherung.at) The authors did not receive any special privileges in accessing the data that other researchers would not have.
